# Vastus lateralis versus rectus femoris muscle flaps for recalcitrant hip joint infection: An anatomical study comparing the effectiveness of acetabular dead space control

**DOI:** 10.1002/ca.23925

**Published:** 2022-07-02

**Authors:** Alexandria H. Smith, Cecilia Brassett, Christopher Gooding, Ahid Abood, Alan Norrish

**Affiliations:** ^1^ Human Anatomy Centre, Department of Physiology, Development and Neuroscience University of Cambridge Cambridge UK; ^2^ Cambridge University Hospitals NHS Foundation Trust Cambridge UK; ^3^ Academic Orthopaedics, Trauma and Sports Medicine University of Nottingham, Queen's Medical Centre Nottingham UK

**Keywords:** Girdlestone procedure, infection, interposition myoplasty, rectus femoris, vastus lateralis

## Abstract

Eliminating recalcitrant prosthetic hip joint infections remains one of the greatest challenges in orthopedic surgery. In such cases, the salvage procedure of femoral head excision (the Girdlestone procedure) is often performed. There has been emerging surgical interest in filling the resulting acetabular dead space with a pedicled muscle flap, to enable antibiotic delivery. Both vastus lateralis (VL) and rectus femoris (RF) muscle flaps have been described for this purpose with good success. This study is the first anatomical investigation comparing VL and RF as candidates for interposition myoplasty following hip joint excision. Following standard surgical technique, the Girdlestone procedure and interposition myoplasty of both RF and VL were performed on 10 cadavers. The primary aim was to determine which muscle flap eliminated a greater volume of acetabular dead space. Secondary aims were to characterize the blood supply to RF and assess additional metrics indicative of the likelihood of flap success. The VL flap eliminated more dead space than RF. However, the use of the RF flap was feasible in all cases and has several benefits, including ease of harvest, mobility, and aesthetics. The location of the inferior vascular pedicle into RF was relatively consistent and the most effective predictor of flap success. Both VL and RF are effective in reducing acetabular dead space. While VL can fill a greater volume, the RF flap has technical advantages, related to the predictability of the blood supply.

## 1 INTRODUCTION

In recent decades, there has been an exponential global rise in the number of primary total hip replacements (THRs) being performed, with approximately 80,000 annually in the United Kingdom alone (Lopez et al., 2017), with a consequent, inevitable rise in prosthetic joint infections. It is estimated that primary THRs carry a 0.6%–2.2% risk of deep prosthetic joint infection (Lenguerrand et al., 2017) resulting in pain, and reduced mobility, necessitating repeated surgery for thousands of patients every year. Tackling hip prosthetic joint infection, particularly recalcitrant infections where revision surgery has failed, remains one of the greatest challenges in orthopedic surgery.

Where a prosthetic joint infection has been present for greater than 6 weeks, either a one‐stage or two‐stage exchange revision can be considered. Where the organism is unknown or has increased virulence, a two‐stage exchange revision is recommended. A one‐stage or two‐stage revision hip arthroplasty successfully eliminates the infection in approximately 90% of patients, leaving 10% of patients with a recalcitrant infection. Further attempts at two‐stage revision may be attempted, but the success rate diminishes. Often patients are left with complex multiorganism infections with multidrug‐resistant organisms. Where no further arthroplasty options can be utilized, the salvage procedure often adopted is excision arthroplasty (Basu et al., 2011; Vincenten et al., 2019). Excision arthroplasty is removal of all foreign material (such as the implants and cement) from the hip joint, leaving the patient without a hip joint (Vincenten et al., 2019). This procedure is often termed the ‘Girdlestone’ procedure, after the surgeon who first described it use for tuberculosis of the hip.

Whilst a simple excision arthroplasty has been used with success for non‐infective conditions such as chronic hip dislocation in patients with non‐ambulatory cerebral palsy (Patel et al., 2015), it is not always successful in eradicating chronic infection of the hip. The most common reason for this that despite adequate debridement at the time of surgery, there will likely be some remaining bacteria in the hip. With a simple Girdlestone excision arthroplasty, the ‘dead space’ in the acetabulum and proximal femur fills with blood and tissue fluid, acting as a culture medium for the remaining bacteria. The tissue in this dead space does not have a blood supply and so does not receive therapeutic doses of antibiotics that are administered in the post‐operative period. In recalcitrant hip infection, control of the dead space with a pedicled muscle flap after debridement is well described (Choa et al., 2010; Lee et al., 1996; Rovere et al., 2021).

The advantage of a muscle flap to fill dead space is that it provides a blood supply to deliver oxygen, leukocytes and antibiotics to the area (Calderon et al., 1986; Feng et al., 1990; Jönsson et al., 1988). Such flaps have also been shown to lower bacterial counts and enhance phagocytic activity of leukocytes in the joint (Eshima et al., 1990). Both rectus femoris (RF) and vastus lateralis (VL) flaps have been described in the literature for this purpose (Alkon et al., 2005; Fischer et al., 2013; Meland et al., 1991; Suda & Heppert, 2010). Whilst a number of studies describe their use, including a systematic review of the evidence (Rovere et al., 2021), to date there have been no anatomical studies to investigate the effectiveness of each of these muscles in eradicating the dead space left following a hip excision.

RF and VL are part of the quadriceps femoris group of muscles in the anterior compartment of the thigh. The predominant function of this muscle group is to extend the knee, with RF having a secondary function of hip flexion. RF receives its blood supply from the descending branch of the lateral femoral circumflex (DBLFC) artery, which arises from the profunda femoris artery, the principal blood supply for the thigh muscles (Williams et al., 2019). The DBLFC artery runs through the intermuscular septum between RF and VL, accompanied by the paired venae comitantes. The DBLFC artery gives off one, two, or three perforators that supply the RF, entering the muscle inferomedially. The most inferior of these perforators forms the dominant blood supply to the muscle and is of particular interest in this study, as it forms the pivot point for rotation of RF in the interposition myoplasty. This same pivot point was used for rotation of the VL muscle flap. The exact position of these perforators has not been fully described in the literature, but they are typically located at around 15 cm inferior to the anterior inferior iliac spine. There are no distal perforators.

The aims of this study are (i) to determine whether RF or VL is the more favorable pedicled flap for interposition myoplasty following hip excision arthroplasty, according to the primary outcome of volume of acetabular dead space eliminated; (ii) to characterize the anatomical variation in the blood supply to RF; and (iii) to assess additional metrics that may be indicative of the likelihood of flap success.

## 2 MATERIALS AND METHODS

**FIGURE 1:**
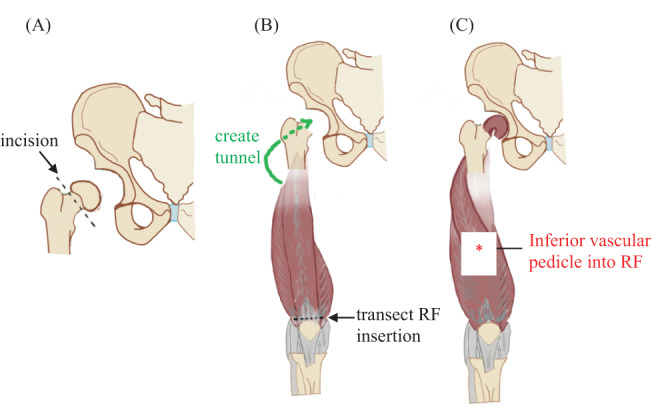
The Girdlestone procedure was used to dislocate and excise the femoral head (A). Interposition myoplasty of RF involved mobilizing it from its insertion onto the patella, creating a subcutaneous tunnel from the anterior compartment of the thigh to the acetabulum (B), and then channeling the RF flap through the tunnel to the acetabulum (C)

A diagrammatic representation of the Girdlestone procedure and interposition myoplasty for RF is shown in Figure 1.

All donors in the Human Anatomy Centre had consented before decease to the use of their bodies for anatomical research, and had been preserved by a vascular embalming method with a solution containing 4.2% formaldehyde via the common carotid or femoral arteries. Donors were collected and stored in compliance with the Human Tissue Act (2004), under Human Tissue Authority License number 12146.

Donors with previous hip replacement operations were excluded from the study. A total of 10 donors were used for this study, with an average age of 82.9 years (range 70–96). There were 5 males and 5 females, all of Caucasian origin.

### 2.1 The Girdlestone procedure

The cadaver was placed in the lateral decubitus position. The lower limb of interest was uppermost. Wooden blocks were used to support the body and the body secured to the table with tape.

**FIGURE 2:**
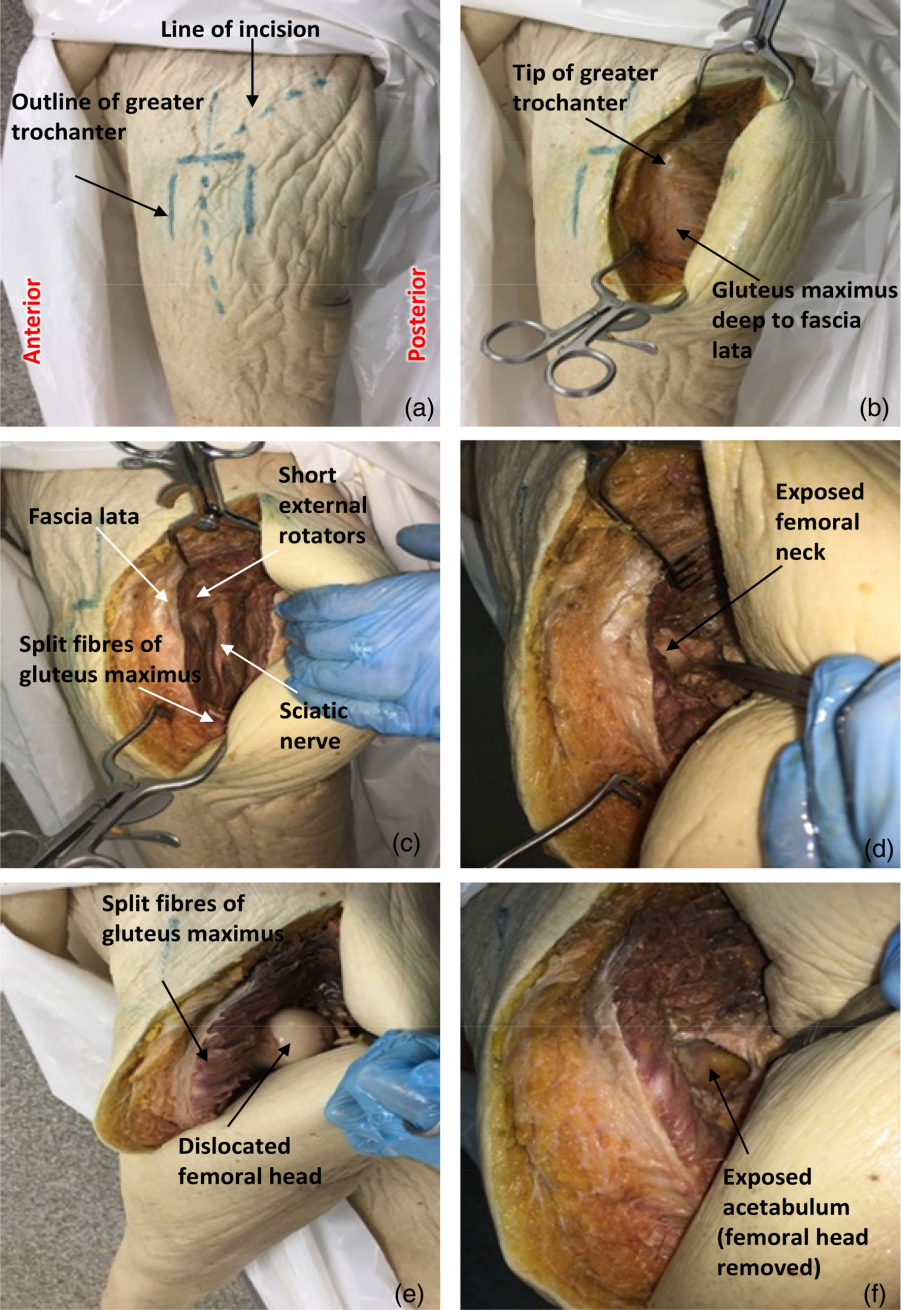
Dissection technique for Girdlestone procedure, with a view of the lateral aspect of the left upper thigh

The outline of the greater trochanter was marked on the skin covering the hip (Figure 2A). A skin incision was made, commencing 12 cm distal to the tip of greater trochanter and centered along the femoral diaphysis. This continued proximally towards the posterior superior iliac spine for 12 cm proximal to the greater trochanter. The underlying subcutaneous fat and, in the distal portion of the incision, the fascia lata (Figure 2B) were also incised along the original line of skin incision. In the proximal portion of the wound, the fibers of gluteus maximus muscle were bluntly divided in a proximal direction, and retracted to reveal the short external rotators.

The sciatic nerve was identified (Figure 2C). The piriformis and short external rotators were transected along the lateral portion of their tendons where they insert onto the greater trochanter. They were then reflected posteromedially to expose the posterior joint capsule and protect the sciatic nerve, as this would have been necessary in clinical practice. The joint capsule was incised to partially expose the femoral neck (Figure 2D).

The femoral head was dislocated by internal rotation and flexion at the hip joint (Figure 2E). The femoral head was then excised by a femoral neck osteotomy using a hammer and chisel (an orthopedic oscillating saw was not available) to expose the acetabulum (Figure 2F).

### 2.2 Preparation for interposition myoplasty

**FIGURE 3:**
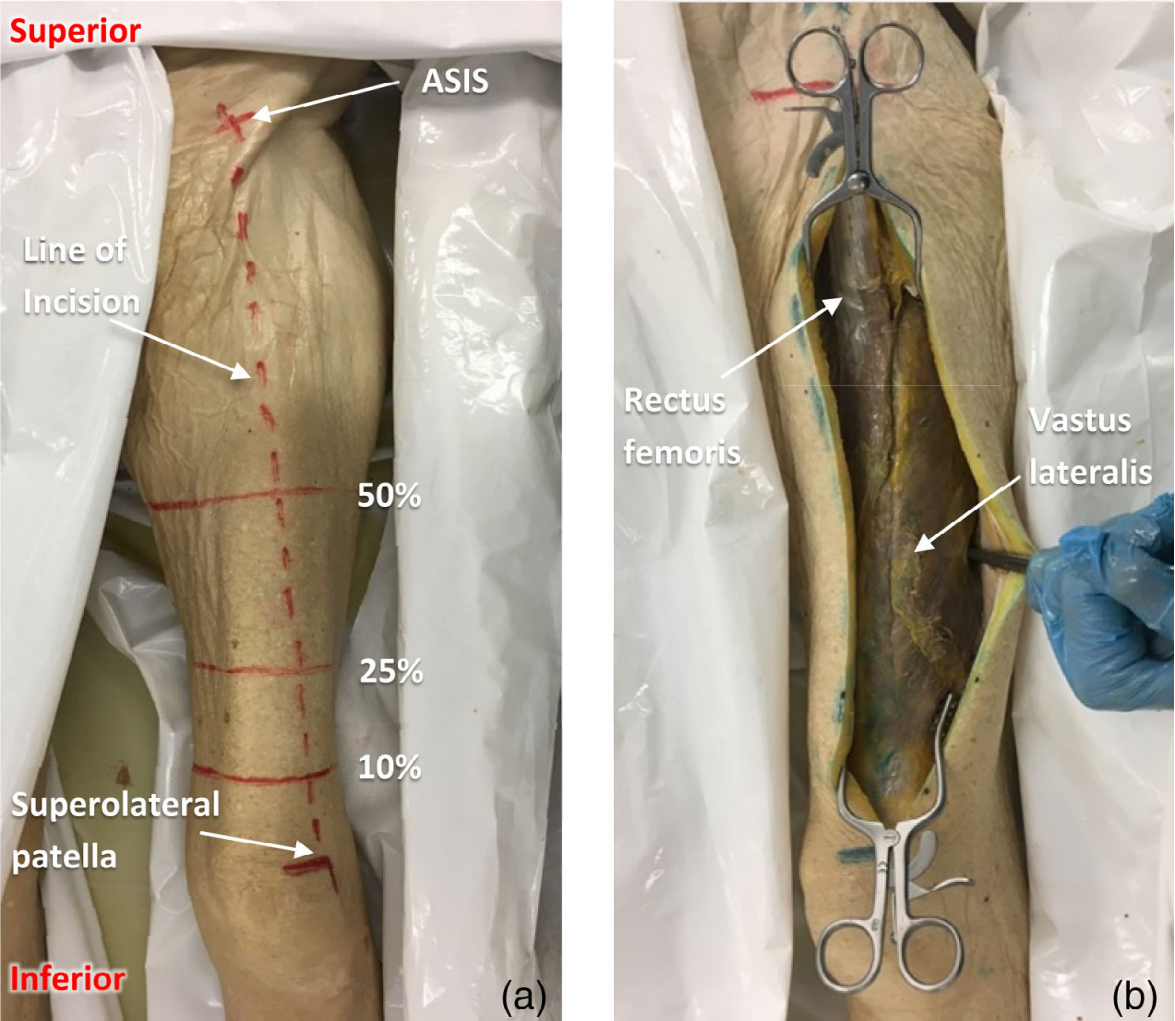
Dissection technique to prepare for the interposition myoplasties, showing the anterior aspect of the left thigh

The donor was placed in the supine position. The surface landmarks of the anterior superior iliac spine (ASIS) and superolateral aspect of the patella were marked out, with a line drawn between them (Figure 3A) denoting the division between RF and VL.

Points were drawn along a line between the ASIS and superolateral patella; these were located at 10%, 25% and 50% of the total distance from the patella (Figure 3A). Thigh circumference was then measured at each of these points.

An incision was made along the surface marking line down to deep fascia. This was opened up medially and laterally. Retractors were used to allow exposure of the anterior compartment (Figure 3B).

### 2.3 Interposition myoplasty: RF

**FIGURE 4:**
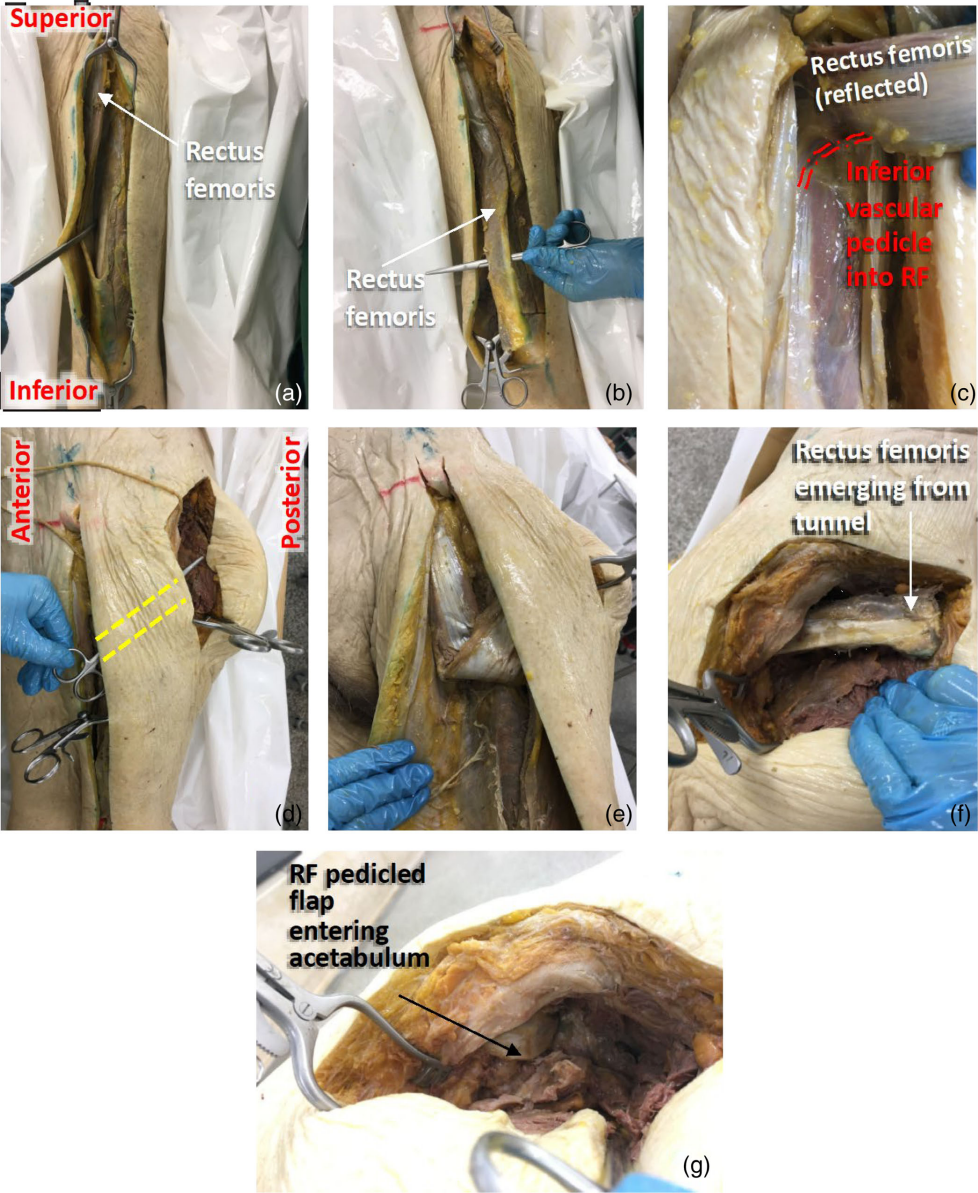
Dissection technique for interposition myoplasty of rectus femoris. (A,B) The anterior aspect of the left thigh. (C) An enlarged view of the dominant artery entering RF. (D) Lateral view of the left thigh, showing the subcutaneous tunnel made from the anterior compartment of the thigh to the acetabulum. (E) An anterior view, showing how the RF flap is rotated and channeled through the tunnel. (F,G) Posterolateral views of the hip, showing the RF flap emerging from the tunnel (F) and then being fed into the acetabular cavity (G)

RF was identified, lifted, and separated from the laterally‐located VL through a plane of loose areolar tissue (Figure 4A). RF was freed from fascial connections on its superficial, deep, and medial surfaces. On its lateral surface, care was taken to protect its vascular pedicle during mobilization. The muscle was freed from its distal attachment by running a finger underneath its distal tendon and transecting it at the point where it fuses to form the quadriceps tendon (Figure 4B). RF was retracted medially to visualize the DBLFC artery and its perforating branches (Figure 4C). The perforators entering the RF were dissected and counted.

A subcutaneous tunnel was made from the anterior compartment of the thigh to the acetabulum by blunt dissection along the shortest possible route (Figure 4D). RF was rotated around its most inferior perforator to ensure minimal tension in the artery (Figure 4E). The pedicled flap was transposed through this tunnel, towards the acetabulum (Figure 4F). It was then placed into the empty acetabulum (Figure 4G). The length of muscle at the end of the flap that enters the acetabulum will be referred to as the ‘RF filler’. RF was then returned to its anatomical position.

### 2.4 Interposition myoplasty: VL

For each cadaver, a VL pedicled flap was also made, to compare with the RF pedicled flap.

**FIGURE 5:**
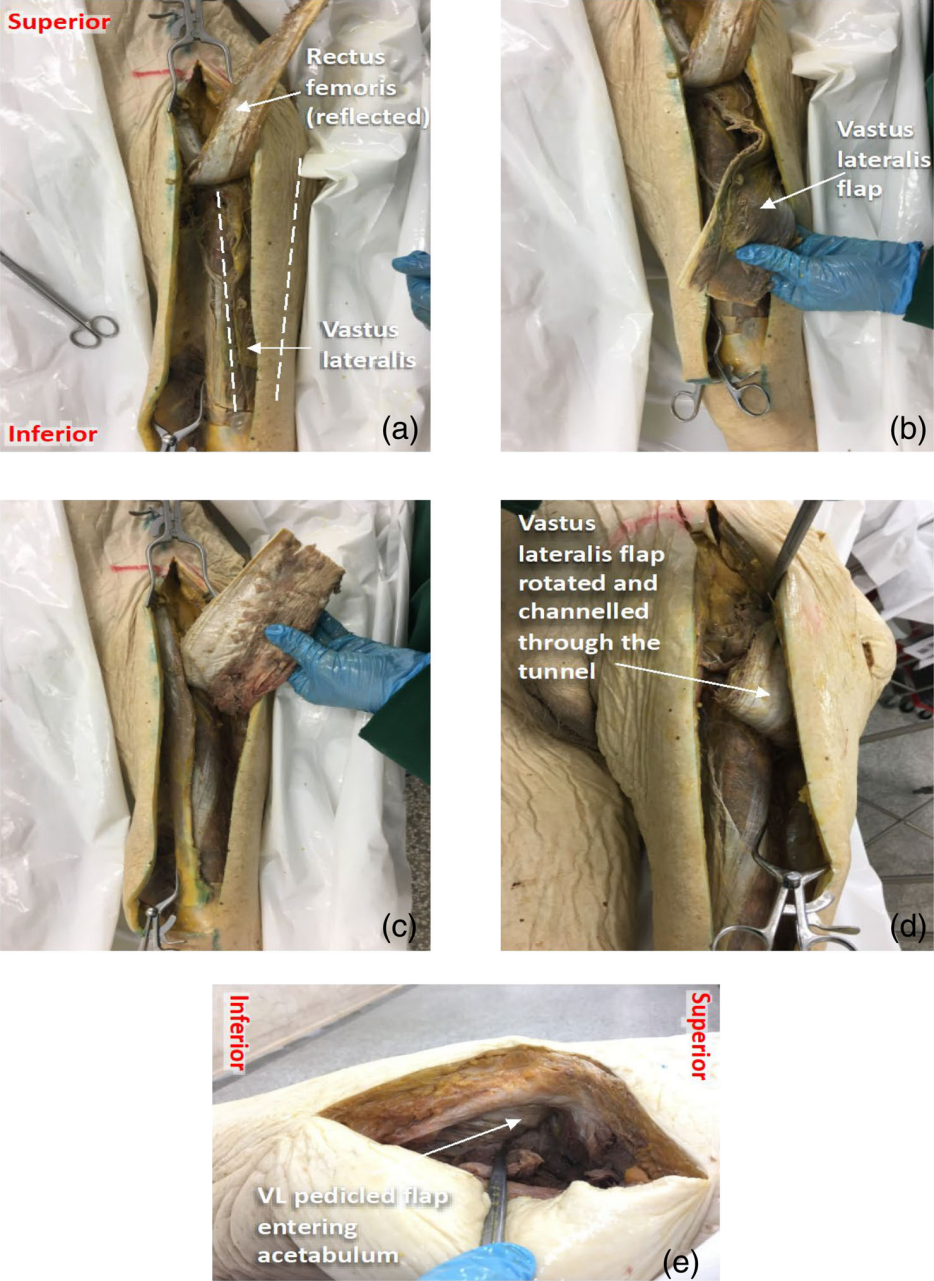
Dissection technique for interposition myoplasty of vastus lateralis. (A–D) Anterior views of the left thigh, showing the VL muscle in situ (A), mobilization of the VL flap (B), rotation of the VL flap (C) and transposition of the flap through the subcutaneous tunnel (D). (E) A posterolateral view of the hip, showing the VL flap entering the acetabular cavity

RF was reflected to expose the underlying VL muscle (Figure 5A). On the anterior aspect of VL, the DBLFC artery with its venae comitantes were located in the subfascial plane. VL was incised along its medial edge to separate it from vastus intermedius, ensuring that the DBLFC artery would be protected and included in the flap. VL was detached from its distal insertion by running a finger along the underside of its tendon and transecting it at the point where it fuses with the quadriceps tendon (Figure 5B). The flap was mobilized proximally by blunt dissection from the fascia along its lateral edge until the muscle could be rotated at the level of the established pivot point, that is, the most inferior artery into RF (Figure 5C).

The resulting VL pedicled flap was transposed through the pre‐made tunnel and into the acetabulum (Figure 5D and E). The length of muscle at the end of the flap that enters the acetabulum will be referred to as the “VL filler.” VL was then returned to its anatomical position.

### 2.5 Determining the length of the RF and VL fillers

Due to the rigidity of the muscles in the cadavers, performing the interposition itself was not considered to be a realistic way of determining the additional muscle length available to fill the acetabular dead space. Instead, various distance measurements were used to determine the length of the filler for RF and for VL, as described in the following text.

**FIGURE 6:**
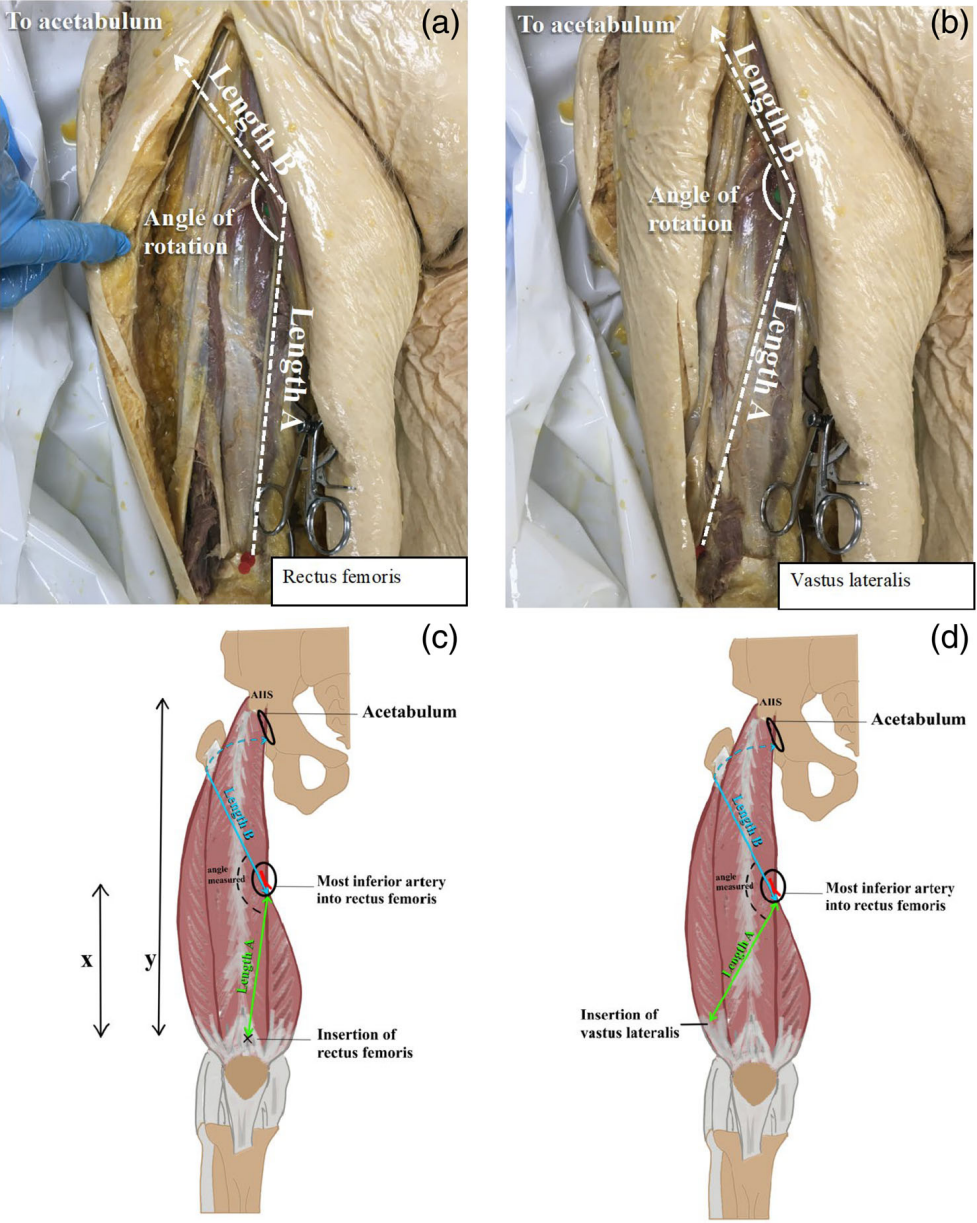
(A, B) Distance measurements used to determine length of filler (length *C*). Length *B* continues to acetabulum. (C, D) The distance measurements (*x* and *y*) used to determine the position of the inferior vascular pedicle

String was used to demarcate the following distances (Figure 6A and C): (a) from the distal insertion of RF to the pivot point, where the inferior vascular pedicle enters RF; and (b) from the pivot point, through the tunnel, to the acetabulum. The string was pulled taut and held in position by pin. To ensure consistency, the string was always pinned to the point on the acetabular rim in line with the ischial tuberosity. The two lengths of string were then measured, with Length A being equivalent to the length of the pedicled flap and Length B the distance of flap used to reach the acetabulum. The length of flap to fill the acetabulum (“filler” or Length C) was calculated as follows:

LengthC=LengthA−LengthB

These length measurements and calculation were repeated for VL (Figure 6B,D).

### 2.6 Measuring the angle of rotation of the flaps in interposition myoplasty

For both RF and VL flaps, the angle of rotation was measured. Lengths of string were positioned as described above. A photo of the angle of rotation (i.e., the angle between the two lengths of string) was taken using a camera held parallel to the floor, centered on the axis of the femoral shaft. A computer program (Mac Application Aequo 1.1, 2019) was used to digitally measure the angle of rotation.

### 2.7 Determining the consistency of the inferior vascular pedicle location

In order to determine whether the position of the inferior vascular pedicle is consistent between different people, its position along the whole length of RF muscle was determined. The thigh incision was continued proximally to trace RF back to its origin on the anterior inferior iliac spine. The distance from the origin of RF to its distal insertion into the quadriceps tendon (distance y in Figure 6C) and the distance from the inferior pedicle to the distal insertion of RF were measured (distance *x* in Figure 6C). The position of the inferior pedicle was then expressed as a proportion of the distance along the RF muscle.

### 2.8 Determining the volume of the RF and VL fillers

Length *C* is the length of the pedicled flap that actually enters the acetabulum. A length of muscle measuring Length *C* was cut off from the distal end of RF. This is referred to as the “RF filler,” that is, the piece of muscle that would occupy the acetabulum to reduce dead space.

The displacement method was used to measure the volume of this piece of muscle. Water was added to a 250 cm^3^ measuring cylinder and the initial volume recorded. The piece of RF tissue was added and the final volume in the measuring cylinder was recorded.

The volume of the piece of RF tissue occupying the acetabulum was calculated as follows:

Volume ofRFfiller=Final volume−Initial volume

This was repeated for VL.

### 2.9 Measuring the volume of the acetabulum

As the femoral head is almost entirely surrounded by the acetabulum at the hip, femoral head volume is a good measure of acetabular volume.

Extraction of the femoral head was performed. The volume of the femoral head was measured by the displacement method. Water was added to a 250 cm^3^ measuring cylinder and the initial volume recorded. The femoral head was added and the final volume in the measuring cylinder was recorded. The femoral head volume, and thus acetabular volume, was calculated as follows:

Femoral head volume=Final volume−Initial volume

### 2.10 Statistical methods

Statistical analysis with graphical representation was performed using GraphPad Prism 8.4 (2020) and Microsoft Excel 16.31 (2019). The data are expressed as the mean ± *SD*. To test if the mean averages of variables were different, paired t‐tests were used. All statistical tests were two‐tailed. *p* Values < 0.05 were considered statistically significant and are indicated by * in the Section 3. p Values < 0.01 are indicated by **. Repeated measures ANOVA testing was used to determine if the sex of the subject had any effect on the percentage of acetabular volume filled by RF and VL.

The parametric significance tests used carried two assumptions: that the data were both normally distributed and homoscedastic. Normal distribution was confirmed by assessing the skew of the data. As this was between 0.8 and 1.2, the data were normally distributed. Homogeneity of variance was assessed using Brown‐Forsythe tests. For data with high heteroscedasticity, such as the measurements of length and volume of muscle filler, analysis was performed using log_10_ transformed data. To test for correlation between two normally distributed variables, simple linear regression was performed. Multiple linear regression was used for the data set of thigh length and acetabular volume to account for sex differences.

## 3 RESULTS

In all 10 cadavers, the RF pedicled flap was sufficiently long to reach the acetabulum and fill some of the dead space.

**FIGURE 7:**
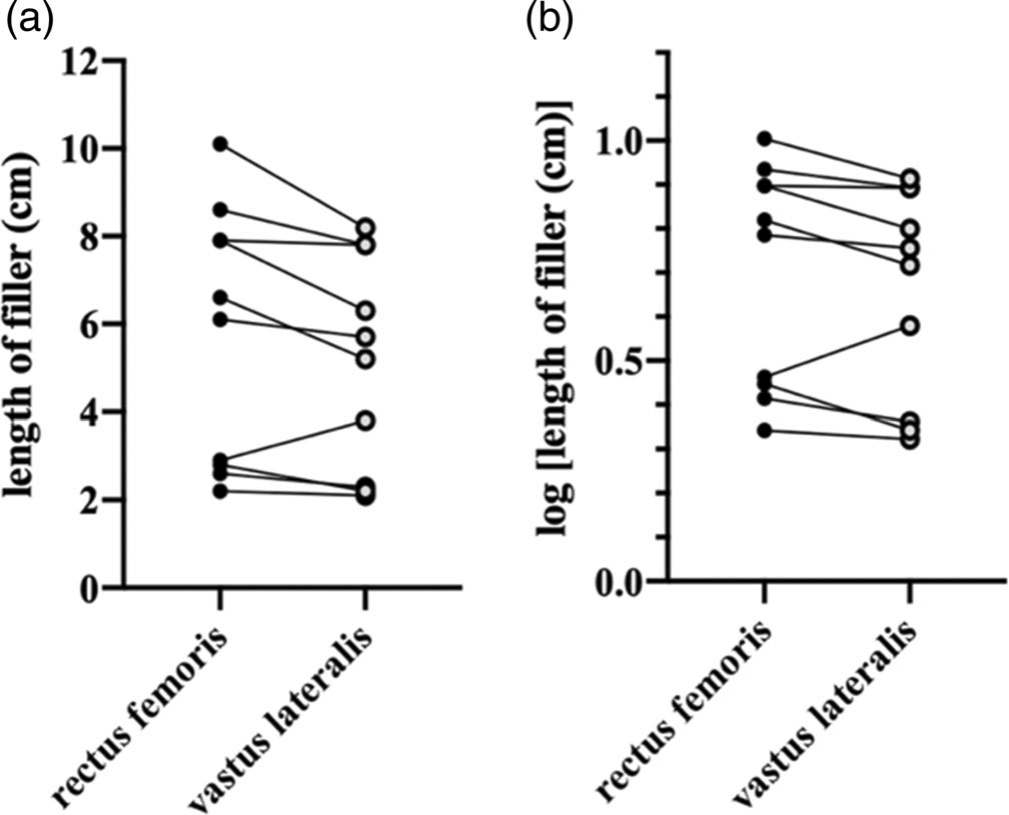
The length of muscle filler for both RF and VL. (A,B) The same data, before and after undergoing log_10_ transformation respectively

In Figure 7A, the difference between the lengths of RF and VL filler within subjects appears multiplicative, rather than additive. The distribution of variance appears better after log_10_ transformation (Figure 7B). Subsequent statistical analysis was therefore performed on the log_10_ data. Within each subject, the length of the RF filler (5.77 ± 2.92 cm) was greater than that for VL (5.14 ± 2.43 cm) (*p* = 0.04*, paired *t*‐test).

**FIGURE 8:**
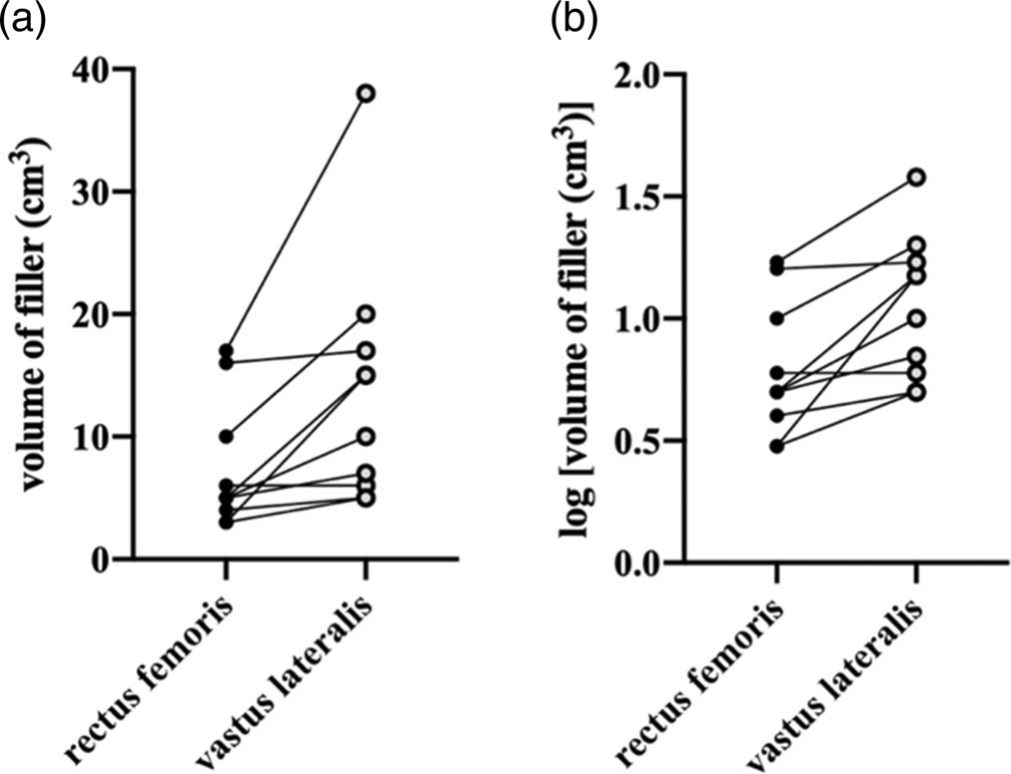
The volume of muscle filler for both RF and VL. (A,B) The same data, before and after undergoing log_10_ transformation respectively

In Figure 8A, the difference between the volumes of RF and VL filler within subjects appears multiplicative, rather than additive. The distribution of variance is improved after log_10_ transformation (Figure 8B). Subsequent statistical analysis was therefore performed on the log_10_ data. Within each subject, the volume of the RF filler (7.40 ± 5.19 cm) was smaller than that for VL (13.80 ± 10.08 cm) (*p* = 0.04*, paired *t*‐test).

**FIGURE 9:**
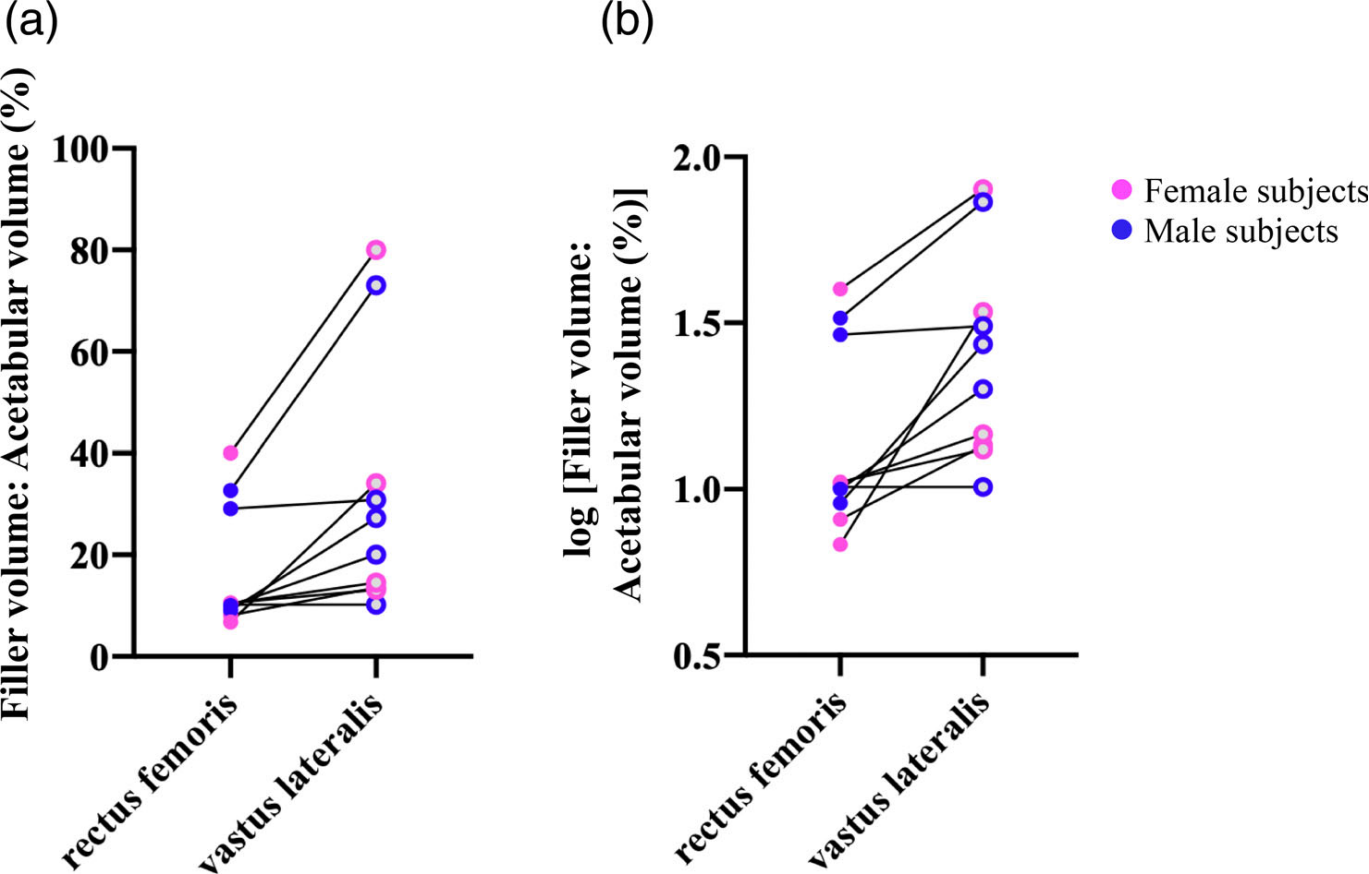
The percentage of acetabular volume occupied by muscle filler for both RF and VL. (A,B) The same data, before and after undergoing log_10_ transformation, respectively

The volume of filler can be expressed as a percentage of the dead space in the acetabulum (Figure 9). One advantage of expressing it this way is that larger individuals, such as males, may have a larger acetabulum, and smaller individuals may have the converse. However, the volume of the muscle filler may also be proportionate to the acetabular size.

The data appear multiplicative in Figure 9A. The distribution of variance appears better after log_10_ transformation (Figure 9B). Subsequent statistical analysis was therefore performed on the log_10_ data. The mean percentage of acetabular volume filled by the RF filler was 16.7% ± 12.2%, and by the VL filler 31.7% ± 25.0% (*p* = 0.004**, paired *t*‐test). A repeated measures ANOVA was carried out to determine if the sex of the subject had any effect on this. There was no difference in the percentage of acetabular volume filled between males and females by either muscle (*p* = 0.67). Sex also had no effect on the difference between RF and VL in terms of the percentage of acetabulum filled (*p* = 0.67).

**FIGURE 10:**
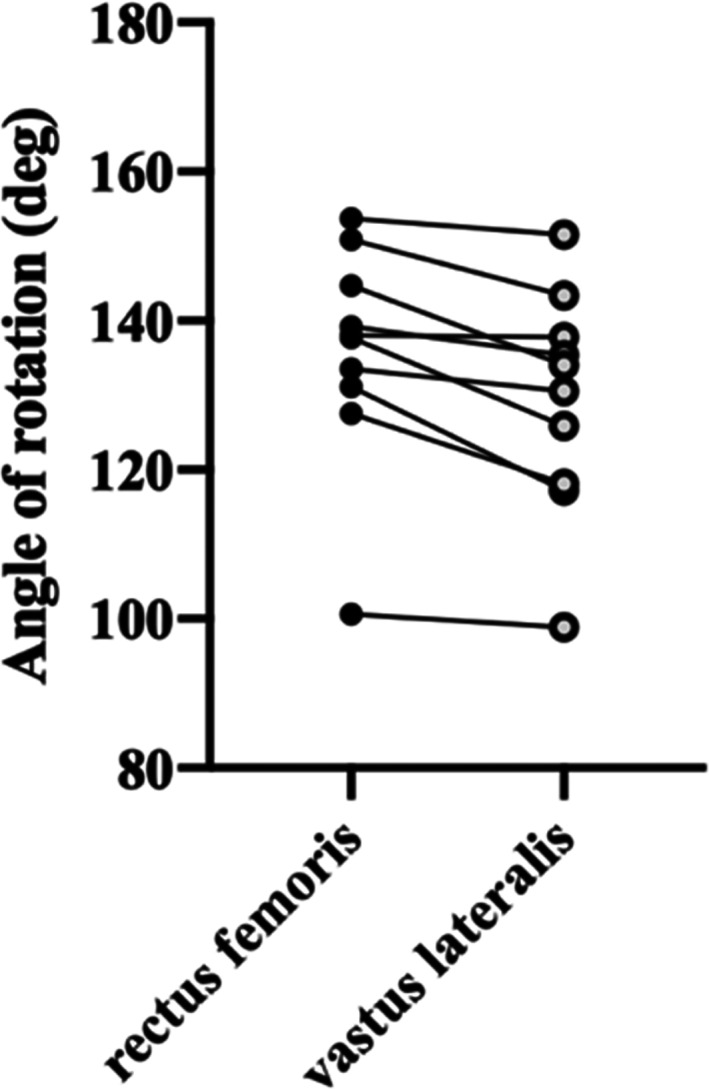
The angle of rotation for the RF and VL pedicled flaps

The mean angle of rotation of the RF flap (135.7 ± 14.8 degrees) was greater than that of the VL flap (129.3° ± 15.0°)(P = 0.002**, paired *t*‐test)(Figure 10).

The mean acetabular volume of the subjects was 46.3 cm^3^ (range 25–59 cm^3^). The mean acetabular volume of males (54.2 ± 3.4 cm^3^) was greater than that of females (38.4 ± 8.7 cm^3^)(*p* = 0.006**, unpaired *t*‐test). As expected, the mean thigh length of males (47.3 ± 2.4 cm) was greater than that of females (42.4 ± 1.9 cm) (*p* = 0.008**, unpaired *t*‐test).

**FIGURE 11:**
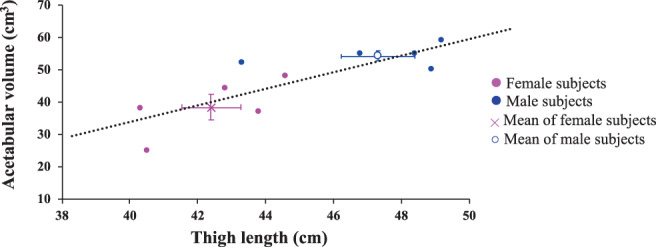
Linear regression of acetabular volume and thigh length. This graph shows the average acetabular volume and thigh length for males and females (mean and *SD*)

Overall, acetabular volume was positively correlated with thigh length (*r* = 0.67, *p* = 0.004**, simple linear regression). However, this may reflect the fact that, on average, women have smaller thighs and acetabular volumes than men (Figure 11). Multiple linear regression was performed to assess the relationship between acetabular volume and thigh length within the male and female groups. This showed that there was no difference in this relationship between the two sexes (P = 0.21, F statistical test).

**FIGURE 12:**
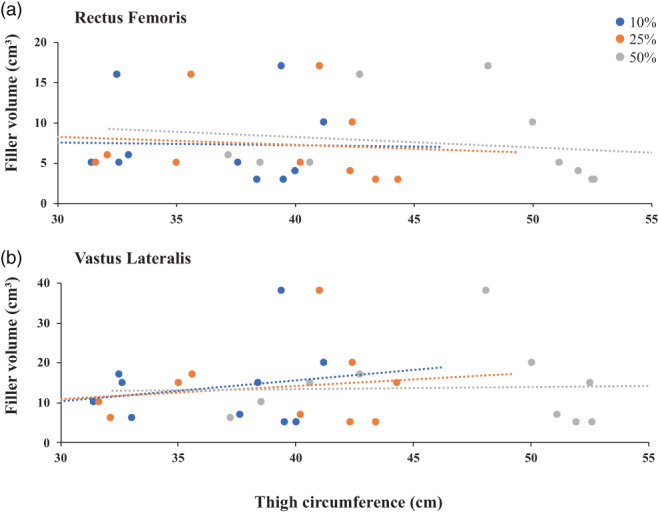
Linear regression of thigh circumference and muscle filler volume for RF (A) and VL (B). Thigh circumferences were measured at various points along the thigh: At 10% (blue), 25% (orange), and 50% (gray) of the distance along the thigh from the suprapatellar margin

**FIGURE 13:**
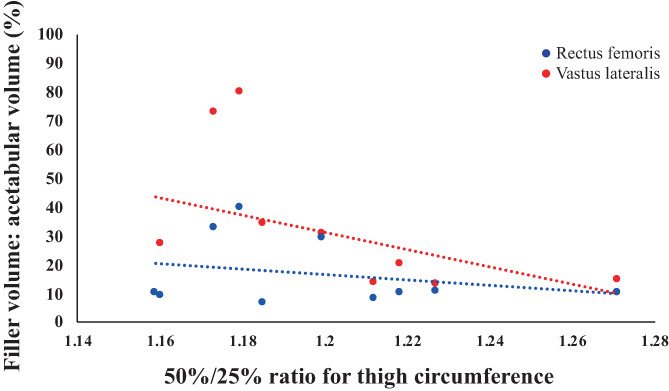
Linear regression of the percentage of acetabulum filled by filler and the 50%/25% ratio for thigh circumference

Simple linear regression of all data revealed no relationship between muscle filler volume and thigh circumference at any of the three points along the thigh (*p* > 0.05 in all cases) (Figure 12). Moreover, thigh morphology is an unconvincing metric for assessing likelihood of flap success. The association between thigh morphology and the percentage of dead space eliminated was investigated. Thigh morphology was defined by the ratio of thigh circumference between the points 50% and 25% of the distance along the thigh (50%/25% ratio). The correlation between the 50%/25% ratio and muscle “filler” volume was very weak (*r* ^2^ = 0.17 for VL, *r* ^2^ = 0.17 for RF, Figure 13). However, the range of thigh circumferences measured was very limited, with all cadavers showing about 20% increase. Simple linear regression analysis was used to assess significance (*p* = 0.24 for VL, *p* = 0.45 for RF, using the *F* statistical test).

Regarding the characterization of the blood supply to RF, 8 subjects had 1 major and 1 minor vascular pedicle; 1 subject had 1 major and no minor pedicles; and 1 subject had 1 major and 2 minor pedicles. All of these pedicles originated from the DBLFC artery.

**FIGURE 14:**
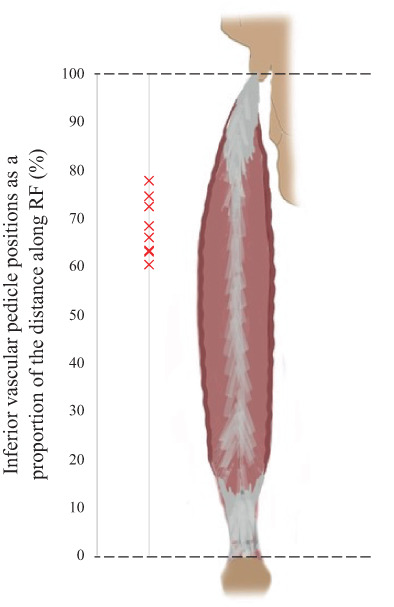
Location of the inferior vascular pedicle, as a proportion of the distance along the length of RF

The most inferior vascular pedicle into RF, which was the pivot point for the muscle flaps, demonstrated a relatively consistent location. The mean distance of the pedicle along the length of RF was 68.3% ± 6.1% from its insertion at the patella. This consistency can be appreciated in Figure 14 and is confirmed by the small coefficient of variation:

$$\text{Coefficient of variation},\mathrm{Cv}=\frac{\mathrm{SD}}{\text{mean}}=\frac{6.1}{68.3}=0.09$$

## 4 DISCUSSION

Recalcitrant prosthetic hip joint infections, following failed revision surgery for infection, are an increasing clinical burden, incurring high costs, along with physical and mental stress on the patient and their healthcare team (Akindolire et al., 2020; Rietbergen et al., 2016). The use of muscle flaps to combat infection and improve the rate of complete eradication of infection is an emerging field in hip revision surgery (Rovere et al., 2021). This cadaveric study has taken an anatomical approach to determine whether RF or VL is more favorable as a donor pedicled muscle flap. In terms of the primary outcome of eliminating dead space in the acetabulum, VL appears to be more favorable than RF. However, there are some benefits to using the RF flap and in certain situations, it could be a valuable addition to the surgical arsenal. The larger the volume of muscle filler (percentage of the acetabulum occupied), the more effective the flap. In all subjects, VL eliminated more dead space than RF, with no difference between males and females. Therefore, VL is the more effective pedicled flap with regards to this outcome measure.

Flaps can fail for several reasons including tension on the vascular pedicle, kinking of the pedicle, vascular thrombosis and persistent infection (Jeffers et al., 2012). The pivot point for rotation of both RF and VL flaps is around the most inferior vascular pedicle into RF. The amount of stress on this artery is dependent on the angle of rotation of the flap around it. In all subjects, the angle of rotation was larger in RF than VL because RF lies in a more medial position. Therefore, RF flaps are less likely to compromise blood flow. However, the size of this effect may be minimal. The mean difference between the angle of rotation for RF and VL is only a few degrees and is close to the standard deviation values.

An additional benefit of the interposition myoplasty procedure is that the flap functions as a mechanical barrier that reduces proximal femoral migration. The flap covers the femoral stump to provide a cushioning effect and prevent painful grinding against the ilium. While a major disadvantage of the procedure is the loss of function of the muscle used, this is not so problematic for non‐ambulatory or paralyzed patients. In addition, mobility would already be very limited following the Girdlestone procedure.

Loss of function of the muscle used for interposition myoplasty can result in weak knee extension and patellar instability, especially in the case of VL, due to the resulting unbalanced force of vastus medialis on the patella. The use of RF as an alternative to VL would potentially lead to less donor site morbidity. A study by Daigeler et al. (2005) found that harvesting of the RF pedicled flap reduced knee extensor strength by up to 20% but did not impact on the range of motion of the knee and hip and was well‐compensated by synergists. Overall, there was high patient satisfaction with the aesthetic and functional outcomes.

Another aspect of this study was to identify metrics available in vivo that could be used to predict the extent of dead space elimination and therefore likelihood of success of the flap. Thigh circumference was investigated because it has been proved to be a good predictor of total cross‐sectional area of the quadriceps (Willan et al., 2002). It was of interest to see if emaciated patients would have a smaller volume of muscle filler. However, the results showed no relationship between muscle filler volume and thigh circumference, measured at 10%, 25%, or 50% along the length of the thigh. Thigh morphology, defined by the ratio of thigh circumference between the points 50% and 25% of the distance along the thigh, does not appear to be an effective predictor of volume of muscle filler. There was only a very weak association between the 50%/25% ratio and volume of filler, for both RF and VL.

This study measured a mean acetabular volume of 46.3 cm^3^ (range 25–59 cm^3^), which corroborates the value stated in the literature: mean 39.8 cm^3^ (range 19.4–60.8 cm^3^) (Jóźwiak et al., 2015). The sexual dimorphism observed also confirms what has been described, i.e. that males have larger femoral head diameters (Kurki, 2011). The position of the inferior vascular pedicle appears to be the most robust predictor of volume of muscle filler. The more proximal the pedicle, the more proximal the pivot point of muscle rotation, and hence the longer the flap, giving a larger section of muscle to act as a filler of the acetabulum. Clinically, vascular doppler ultrasound could be used to locate the perforator pre‐operatively to determine the likelihood of success for a pedicled flap.

A secondary aim of this study was to determine the consistency of the location of the inferior vascular pedicle into RF. Figure 14 shows that the inferior pedicles across the 10 subjects were clustered in a zone at 60%–77% of the distance along the length of RF. There was a small coefficient of variation in its location between subjects. This consistency is useful for surgeons mapping out the likely location of the pedicle before performing interposition myoplasty. Anatomical variations in blood supply to RF largely lie in the number of pedicles entering the muscle. These perforating arteries are located close together and likely form anastomotic connections. Therefore, if one of the arteries were compromised by interposition myoplasty, the other(s) may be able to compensate. Pre‐operative determination of the number of pedicles may be useful to assess the risk of vascular compromise.

This study has some limitations. The embalming process for cadavers causes some shrinkage and hardening of tissues, which may have altered the morphology and volume of the muscles studied. As all cadavers had been subject to the same process, RF and VL were assumed to have been equally affected. Moreover, the rigidity of the muscle flaps reduced their mobility, which made the interposition myoplasty difficult to perform, in contrast to clinical practice. Therefore, the length of muscle flap was determined using various length measurements in situ, rather than from the interposition itself. However, the dissections themselves followed the correct surgical procedures to keep them as realistic as possible. A larger sample size would increase the power of statistical analysis. An equal number of male and female cadavers were used. All subjects were elderly; this group is clinically relevant because the average age of patients requiring THR is 65 years (Passias & Bono, 2006). As all subjects were Caucasian, these results may not be applicable to the wider population. An additional demographic that could not be obtained in this study was the living height and weight of the subjects, which may explain some of the variation in the volumes of muscle filler and acetabulum.

This study has demonstrated the superiority of the VL flap in filling the acetabular dead space, but has also demonstrated the feasibility and some potential advantages of using the RF flap: (i) its reliable anatomy, (ii) robust blood supply, (iii) ease of harvest, (iv) mobility, and (v) aesthetics. The location of the inferior vascular pedicle into RF is the most effective predictor of flap success and is shown to be relatively consistent.

Further clinical studies could involve an in vivo assessment of donor‐site morbidity. Gait analysis could be used to compare the functional loss in RF and VL myoplasty. Other future directions for clinical assessment include flap aesthetics, effectiveness in relieving pain and success in eradicating infection.

## 5 CONCLUSION

The VL muscle flap is more favorable than RF for filling acetabular dead space after excision arthroplasty of the hip. However, RF is a feasible and valuable alternative with some important advantages.
